# Effect of feed trace elements on eggs of five strains of laying hens and their health risk assessment

**DOI:** 10.1002/vms3.1184

**Published:** 2023-06-11

**Authors:** Mohammad Hashemi, Amin Azadi, Masumeh Saghi, Majid Aminzare, Seyyed Mohammad Ali Noori

**Affiliations:** ^1^ Medical Toxicology Research Center Mashhad University of Medical Sciences Mashhad Iran; ^2^ Department of Nutrition, Faculty of Medicine Mashhad University of Medical Sciences Mashhad Iran; ^3^ Department of Food and Drug Control, Faculty of Pharmacy Ahvaz Jundishapur University of Medical Sciences Ahvaz Iran; ^4^ Student Research Committee, Department of Environmental Health Engineering, School of Health Mashhad University of Medical Sciences Mashhad Iran; ^5^ Department of Food Safety and Hygiene, School of Public Health Zanjan University of Medical Sciences Zanjan Iran; ^6^ Toxicology Research Center Medical Basic Sciences Research Institute Ahvaz Jundishapur University of Medical Sciences Ahvaz Iran

**Keywords:** ICP–OES, iron, micronutrients, total hazardous quotient, zinc, chicken egg

## Abstract

**Background:**

Trace elements are essential for human nutrition, and their deficiencies or excesses are strongly associated with several diseases, such as cardiovascular ones.

**Objectives:**

The current cross‐sectional study investigated the concentration of essential trace elements (copper, non‐metal selenium, iron, zinc, cobalt and manganese) in eggs and diets of five strains of laying hens.

**Methods:**

The yolk and albumen were analysed separately, and wet preparation was carried out following inductively coupled plasma–optical emission spectrometry detection. The target hazard quotients (THQs) for the non‐carcinogenic disease were calculated by the United States environmental protection agency (USEPA) method.

**Results:**

The highest concentrations of selenium, zinc and manganese were found in egg yolks of native hens (0.76, 44.22 and 6.52 mg/kg, respectively). The highest amounts of copper and cobalt were recorded in the egg yolk of Lohman (2.07 and 0.023 mg/kg, respectively). On the other hand, the egg yolk of Bovans contained the highest amount of iron (57.46 mg/kg).

**Conclusion:**

Overall, the potential health risks were minimal, and the consumption of eggs was generally safe.

## Graphical abstract

Graphical Abstract: Analysis of Cu, Co, Mn, Zn and Fe in egg samples.

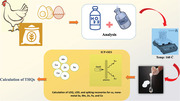

## 1 INTRODUCTION

Copper (Cu), manganese (Mn), iron (Fe), zinc (Zn), cobalt (Co) and non‐metal selenium (Se) are known as ‘trace elements’ that are necessary for human growth and development. These trace elements belong to micronutrients and minor quantities of them (usually less than 100 mg/day) are essential for humans to support their health (Alzahrani et al., 2017). Cu and Mn are necessary for the development and function of bones. Cu participates in energy metabolism, enzyme action and nervous system development. Co is a part of vitamin B12, non‐metal selenium and Fe are well known as antioxidants, but iron sometimes plays a role as a pro‐antioxidant, too (Alzahrani et al., 2017). Furthermore, some diseases like cancer, cardiovascular diseases and diabetes have also been associated with trace elements (Fraga, 2005; Yang et al., 2022).

Insufficient or excessive amounts of trace elements are both harmful. Moreover, essential metals can be accumulated in organs, if excessive quantities enter the body. This does not only happen in humans but also avians. Avians also need trace elements like Cu, non‐metal Se, Zn, Fe, Co, Mn, chromium (Cr), lithium (Li), molybdenum (Mo) and nickel (Ni) for their normal development (Noetzold et al., 2022). Polluted feed, water, environmental factors like outdoor and indoor air, veterinary medicines and mineral supplements may transfer excessive amounts of trace elements into eggs (Dobrzański et al., 2007; Vincevica‐Gaile et al., 2013).

On the other hand, the egg is a high‐quality foodstuff, and in comparison with other protein sources, it is of lower cost. The egg consists of a shell, membrane shells, albumen and yolk (Yao et al., 2014). The egg is consisted of shell, membrane shells, albumen and yolk (Yao et al., 2014). It is also regarded as a vital source of non‐metal Se in countries like Japan, Portugal and Greece (Pinto et al., 2016). At the same time, it contains a variety of organic compounds such as proteins and lipids, with the ability to bind trace elements ions like ovalbumin (an egg white protein), which chelates with Zn, Cu and Mn, or egg yolk lipids, which chelate with Cr, Zn, Ni (Vincevica‐Gaile et al., 2013).

The chicken strain could influence egg composition, size, shell thickness and weight (Pinto et al., 2016; Scott & Silversides, 2000; Yao et al., 2014). Recently, many studies focused on the productive performance parameters (feed intake, egg production, shell thickness, egg weight, shell weight etc.) of hens among different strains (Ershad, 2005; Scheideler et al., 1998; Scott & Silversides, 2000; Singh et al., 2009) and diet or concentration of trace elements of eggs (Abdulkhaliq et al., 2012; Ali et al., 2016; Bologa et al., 2014; Demirulus, 2013; Korish & Attia, 2020; Uluozlu et al., 2009; Vincevica‐Gaile et al., 2013; Waegeneers et al., 2009). However, there is no study focusing on the impact of laying hens strains on the concentration of micronutrients in the egg. Thus, the current study was conducted to examine: (1) the concentration of essential trace elements (Cu, none‐metal Se, Fe, Zn, Co and Mn) of eggs and diets in five strains, including Hy‐Line W36, Shaver White, Lohman LSL‐Lite, native hens (*Gallus gallus* domesticus) and Bovans White; (2) the association among the strain, the diet and the concentration of essential metals in the eggs; (3) the none‐carcinogenic health risk assessment via the consumption of egg.

## 2 MATERIALS AND METHODS

### 2.1 Sample collection

Five strains in cages system farms offering different commercial feeds were examined, including Hy‐line W36, Shaver White, Lohman LSL‐Lite, Bovans White and Native laying hens of Khuzestan and Razavi Khorasan province (*G. gallus* domesticus). A total of 65 samples were analysed, including 50 unfertilized egg samples (10 samples per strain without sacrificing), and 15 diet samples (3 samples per strain). In eggs, yolk and white were analysed separately. The samples were obtained from poultry farms from February to June 2019. Samples (eggs and feeds) were transferred to the central laboratory, and analytical procedures were carried out immediately. Chemically stable glass and plastic tools were used to avoid possible chemical contamination. Before use, all the glassware and plastics were rinsed with diluted HNO_3_ (1:9) and then with distilled water (Pinto et al., 2016; Vincevica‐Gaile et al., 2013).

### 2.2 Egg and feed analyses

Egg samples were kept at 5°C until analysis. The egg white, egg yolk and feed samples were prepared by a method previously described by Vincevica‐Gaile et al. (Vincevica‐Gaile et al., 2013). First, the separation of yolk and albumen was carried out carefully and poured into a weighed plastic container. Then 5 ± 0.01 g of samples were weighed on an analytical balance in glass containers. A volume of 5 mL of H_2_O_2_ (Merck, Darmstadt, Germany) and 10 mL of HNO_3_ 65% (Merck, Darmstadt, Germany) were added to the mixture and held overnight. Then, the mixtures were heated at 160°C using a heater (IKA C‐MAG HS10‐Germany) to obtain pure solutions, and round clock glasses were used to avoid the vaporization of trace elements. The final solutions were then filtered into labelled 50 mL volumetric flask with ash‐less Whatman papers (Vincevica‐Gaile et al., 2013).

### 2.3 Apparatus and trace element detection

The toxic trace elements: Cu, none‐metal Se, Mn, Zn, Fe and Co in feeds and eggs were determined using inductively coupled plasma–optical emission spectrometry (ICP–OES) (SPECTRO‐ARCOS, GERMANY). The operation parameters for detection were 1 L/min nebulizer flow and 2.5 mm fixed plasma torch (Table 1). Multi‐element standard solutions (Carlo Erba reagents) were applied to setup calibration curves. For every 10 samples, 1 blank sample was supplied. The limit of quantification and the limit of detection (LOD), as well as spiking recoveries, shown in Table 2, were calculated for each trace element. Recoveries were nearly quantitative, varying from 82% to 103% in all egg and feed samples.

**TABLE 1:** Operation parameters of inductively coupled plasma–optical emission spectrometry (ICP–OES) instrument.

Instrument	SPECTRO ARCOS
Replicates	3
Spray chamber	Scott
Measure time parameter (s)	28
Auxiliary flow (L/min)	0.060
Coolant flow (L/min	12
Global preflush parameter (rpm)	Fast speed 63, normal speed 30
Pump speed (rpm)	25

**TABLE 2:** Limit of detection (LOD), limit of quantification (LOQ) and recoveries for white, yolk and feed.

Metal	Cu	Se	Fe	Zn	Co	Mn
LOD (ppb)	2	4	2		2	1
LOQ (ppb)	6	12	6		6	3
White spike recovery (%)	102.20	92.44	102.21	98.69	93.09	99.51
Yolk spike recovery (%)	99.81	95.97	99.42	100.10	100.68	97.50
Feed spike recovery (%)	99.27	82.37	100.50	100.37	95.28	99.27

**TABLE 3:** Mean concentrations of essential heavy metals, standard deviations, median, interquartile range for egg white in fives trains of poultry hens.

White egg	Type	Mean (mg/kg)	Std. deviation	Median	Interquartile range	*p* Value
Cu	Bovans	0.303	0.193	0.228	0.094	
	Hi‐line	0.063	0.045	0.048	0.017	
	LSL	0.811	0.290	0.710	0.365	<0.001
	Native	0.394	0.287	0.307	0.289	
	Shaver	0.441	0.207	0.342	0.369	
Se	Bovans	0.444	0.954	0.153	0.050	
	Hi‐line	0.038	0.004	0.038	0.006	
	LSL	0.195	0.074	0.224	0.183	<0.001
	Native	0.227	0.113	0.256	0.194	
	Shaver	0.331	0.045	0.326	0.091	
Fe	Bovans	12.052	4.355	10.675	6.088	
	Hi‐line	0.335	0.132	0.312	0.225	
	LSL	13.384	3.405	13.860	5.320	<0.001
	Native	23.408	3.317	10.823	6.824	
	Shaver	5.290	3.846	3.788	5.247	
Zn	Bovans	7.770	1.680	7.136	1.199	
	Hi‐line	0.346	0.022	0.336	0.036	
	LSL	9.130	2.119	9.072	1.644	<0.001
	Native	21.909	40.550	7.331	12.885	
	Shaver	6.674	6.986	3.364	5.272	
Co	Bovans	0.003	0.003	0.001	0.002	
	Hi‐line	0.001	0.000	0.001	0.000	
	LSL	0.019	0.027	0.010	0.012	<0.001
	Native	0.012	0.013	0.008	0.015	
	Shaver	−0.004^a^	0.008	0.001	0.015	
Mn	Bovans	1.378	0.529	1.294	0.211	
	Hi‐line	0.035	0.022	0.030	0.004	
	LSL	1.423	0.233	1.411	0.295	<0.001
	Native	3.514	5.887	1.302	2.380	
	Shaver	0.763	1.024	0.315	0.844	

^a^ Not detected.

### 2.4 Egg health risk assessment

target hazard quotients (THQs) were calculated for the non‐carcinogenic diseases by United States environmental protection agency (USEPA) method as the following equation (Means, 1989): THQ = [(EF × ED × FIR × C)/(RfD × WAB × TA)] × 10‐3, where EF is exposure frequency (356 days/year), ED is the exposure duration (70 years for adults, 14 years for children), FIR is the food consumption rate (g/person/day), and C is the metal concentration in egg white and yolk (basis on the fresh weight) obtained from the current study (mg/kg); RfD shows oral reference dose, WAB is the average body weight in adults and children according to Shaheen et al. (2016), and TA is the average exposure time for non‐carcinogens (365 days/year × number of exposure duration 70 years). The standard RfD values based on USEPA are Cu = 0.04, Zn = 0.03 mg/kg bw/day. When the THQ values are below one, the resident people will experience no potential health risk due to ingesting the product (Shaheen et al., 2016). Iranian daily egg consumption equals one egg/day/capita. However, in the current study, the own weight of each egg's white and yolk was used instead of the standard weight of an egg (60 g). The mean body weights of an adult and child (60 and 27 kg, respectively) were obtained from previous literature (Ki et al., 2017; Wang et al., 2005).

### 2.5 Statistical analysis

The sample size was calculated using a confidence level of 95%. The mean concentration of the metals was provided according to the mean frequency weights of the white and yolk. When the trace element concentration was below the instrument detection limit, mean LOD was inputted in the analysis (Ali et al., 2016). Pure data were entered into the regression analysis to take the regression coefficient of strain, feed, weight and white/yolk on the heavy metal transition to egg samples. In this study, regression analysis was used because it shows the negative and positive effect of each stain on each metal concentration, not just a total effect of strain as a number. The one‐sample mean‐comparison test was used to compare the standard values. Moreover, the pairwise correlation was performed to study the association in metals in white, yolk and feed separately. Statistical analysis was carried out using SPSS 16 software (*p* < 0.05).

## 3 RESULTS AND DISCUSSION

### 3.1 Effect of feed and strain on micronutrients concentration in egg

**Cu**: As presented in Tables 3 and 4, the highest concentration of Cu was found in the egg yolk of the LSL strain (2.074 mg/kg) and the lowest concentration in the egg white of the Hi‐line strain (0.063 mg/kg). In the study of Bologa et al. (2014), the Cu values in total egg were 1.23–1.34 mg/kg wet weight, comparable to values found in this study. The mean Cu content of egg yolk was higher than that in the egg white for all the strains. The mean range of Cu content in the study of Salar‐Amoli and Ali‐Esfahani (2015) was 1.110–9.510 mg/kg, which was higher than the data obtained by this study. These discrepancies could be attributed to the different sampling procedures, sampling area and diet. The strain had a significant impact on egg Cu content. However, in the study of Salar‐Amoli and Ali‐Esfahani (2015), the effects of strains were negligible. Cu concentration in the diet ranged from 6.82 mg/kg in the LSL strain to 17.04 mg/kg in the Hi‐line strain (Table 5). Moreover, the Cu content in the diet of the Lohman Brown in the study of Salar‐Amoli et al. (Salar‐Amoli & Ali‐Esfahani, 2015) was equal to 18.60 mg/kg, values higher than that reported in the present study. It should be noted that Cu feed content significantly affected (*p* < 0.001) egg Cu content in the five strains (Table 5).

**TABLE 4:** Mean concentration of essential heavy metals, standard deviations, median, interquartile range for egg yolk in five strains of poultry hens.

Yolk egg	Type	Mean (mg/kg)	Std. deviation	Median	Interquartile range	*p* Value
Cu	Bovans	1.462	0.181	1.477	0.299	
	Hi‐line	0.823	0.995	0.207	1.104	
	LSL	2.074	0.868	1.868	0.634	<0.001
	Native	1.192	0.467	1.181	0.758	
	Shaver	1.536	0.545	1.479	0.739	
Se	Bovans	0.425	0.131	0.383	0.098	
	Hi‐line	0.311	0.300	0.087	0.586	
	LSL	0.562	0.193	0.540	0.316	<0.001
	Native	0.763	0.315	0.814	0.513	
	Shaver	0.730	0.190	0.670	0.128	
Fe	Bovans	57.468	8.486	57.191	9.601	
	Hi‐line	20.283	21.265	4.966	40.475	
	LSL	51.272	10.789	50.512	11.098	<0.001
	Native	24.700	11.649	23.648	10.833	
	Shaver	54.615	13.720	51.564	13.929	
Zn	Bovans	37.633	3.934	39.021	7.709	
	Hi‐line	15.321	15.556	3.771	29.913	
	LSL	37.610	8.752	34.223	10.722	<0.001
	Native	44.225	41.310	33.487	24.432	
	Shaver	38.588	13.438	31.961	15.090	
Co	Bovans	0.004	0.004	0.002	0.005	
	Hi‐line	−0.007^a^	0.010	0.001	0.019	
	LSL	0.023	0.025	0.017	0.010	<0.001
	Native	0.014	0.011	0.009	0.014	
	Shaver	−0.052^a^	0.161	0.001	0.004	
Mn	Bovans	4.635	0.487	4.717	0.966	
	Hi‐line	1.665	1.700	0.409	3.218	
	LSL	4.685	1.168	4.251	1.615	<0.001
	Native	6.519	6.274	4.641	3.386	
	Shaver	4.293	1.655	3.651	2.092	

^a^ Not detected.

**TABLE 5:** The number of mean concentrations of trace elements for feed in five types of poultry hens.

Type		Cu (mg/kg)	Se (mg/kg)	Fe (mg/kg)	Zn (mg/kg)	Co (mg/kg)	Mn (mg/kg)
Bovans	Feed	12.88 ± 0.054	1.28 ± 0.102	230.36 ± 5.332	115.57 ± 4.554	0.14 ± 0.001	128.0 ± 0.288
	Egg	0.88 ± 0.62	0.17 ± 0.66	34.76 ± 24.21	22.70 ± 15.60	0.001 ± 0.01	3.00 ± 1.74
	*p* Value	<0.001	<0.001	<0.001	<0.001	<0.001	<0.001
Hi‐line	Feed	17.04 ± 5.274	0.21 ± 0.320	179.15 ± 10.036	105.50 ± 11.234	0.34 ± 0.155	141.05 ± 4.739
	Egg	0.44 ± 0.79	0.17 ± 0.25	10.31 ± 17.86	7.83 ± 13.18	−0.005 ± 0.01	0.85 ± 1.44
	*p* Value	<0.001	0.77	<0.001	<0.001	<0.001	<0.001
LSL	Feed	6.82 ± 0.760	0.26 ± 0.395	178.17 ± 25.021	138.03 ± 22.851	0.13 ± 0.017	150.13 ± 13.567
	Egg	1.44 ± 0.90	0.38 ± 0.24	32.33 ± 20.94	23.37 ± 15.87	0.02 ± 0.03	3.05 ± 1.86
	*p* Value	<0.001	0.37	<0.001	<0.001	<0.001	<0.001
Native	Feed	11.24 ± 0.063	1.43 ± 0.012	143.02 ± 2.298	52.53 ± 0.332	0.08 ± 0.003	110.66 ± 2.015
	Egg	0.79 ± 0.56	0.49 ± 0.36	24.05 ± 17.95	33.07 ± 41.45	0.01 ± 0.01	5.02 ± 6.12
	*p* Value	<0.001	<0.001	<0.001	0.05	<0.001	<0.001
Shaver	Feed	13.38 ± 4.836	0.37 ± 0.288	161.70 ± 3.491	100.82 ± 5.752	0.08 ± 0.021	124.11 ± 3.108
	Egg	0.99 ± 0.69	0.53 ± 0.24	29.95 ± 27.14	22.63 ± 19.41	−0.03 ± 0.11	2.53 ± 2.25
	*p* Value	<0.001	0.23	<0.001	<0.001	0.02	<0.001

**None‐metal Se**: Approximately 80% of ingested non‐metal Se will be absorbed in the human gastrointestinal tract. The non‐metal content of Se in foods depends on geographical locations, and it is necessary to monitor it regularly in soil and foods (Pinto et al., 2016). The none‐metal Se had the lowest mean concentration after Co ranging from 0.038 mg/kg in the egg white of Hi‐line strain to 0.763 mg/kg in the egg yolk of the native strain (Tables 3 and 4). There were almost equal concentrations of non‐metal Se in the white and yolk of the Bovans strain. Many studies reported a higher content of non‐metal Se in yolk compared to the white in different strains (Dobrzański et al., 2007; Giannenas et al., 2009; Gjorgovska et al., 2012). There were significant differences in non‐metal Se amounts only between Bovans and Native feed samples (Table 5). Giannenas et al. (2009) and Gjorgovska et al. (2012) reported a significant effect of dietary non‐metal Se on egg non‐metal Se deposition. Non‐metal Se dietary content in Lohman LSL‐Lite in the current study was 0.26 mg/kg, which is comparable with the reported non‐metal Se content (0.32 mg/kg) in the standard diet of Lohman Brown (Dobrzański et al., 2007).

**Fe**: Fe content in egg yolk and white showed a significant variation among different strains. Fe concentration ranged from 0.335 mg/kg in the egg white of the Hi‐line strain to 57.468 mg/kg in the egg yolk of the Bovans strain (Tables 3 and 4). Chowdhury et al. (2011) reported values ranging from 2.96 to 27.27 mg/kg in eggs. Fe also had the highest concentration among the tested essential metals in the diet (Table 5). Therefore, it may be considered that the major source of Fe in the eggs is diet, as the former studies in the literature reported (Durge et al., 2022; Vincevica‐Gaile et al., 2013). Fe content in the feed of selected strains ranged from 143.02 mg/kg in the native strain to 230.36 in the Bovans strain (Table 5).

**Zn**: Zn content had the second highest concentration in the eggs, and it was in the range of 0.346 mg/kg in egg white in Hi‐line (Table 3) and 44.225 mg/kg in egg yolk in the native strain (Tables 4). Apart from Hi‐line, the native strain had the highest Zn content in white (21.909 mg/kg) and yolk (44.225 mg/kg). As previously stated, the strain could have a significant effect on egg trace element content. It seems that the effect of strain is more probable than diet in the case of Zn (Table 5). The data obtained by the current study (Tables 3 and 4) were inconsistent with the work of Dermilus et al., which reported 6.2 mg/kg in albumen and 43.9 mg/kg in the yolk, indicating that Zn values are higher in egg yolk than white (Demirulus, 2013). Bologa et al. (2014) found less Zn, ranging from 1.23 mg/kg wet weight to 1.49 mg/kg wet weight, which may be due to differences in the selected strain, the production systems (organic vs. conventional), or environmental factors such as the water and the feed.

**Co**: The Co was not detected by the ICP–OES instrument (2 ppb, Table 2), especially in Shaver (Table 3) and Hi‐line strains (Table 4). The lowest and the highest concentration of Co were found in the egg white of Hi‐line (0.001 mg/kg fresh weight) and the egg yolk of LSL (0.023 mg/kg fresh weight), respectively. Chowdhury et al. (2011) revealed that Co concentration in samples of egg white was below the detection limit in farm hens, as well. Song et al. (2006) stated that the Co content ranged between 4.13 and 4.37 μg/kg in the egg samples. Moreover, a major difference in the Co content of eggs in different strains was observed (Tables 3 and 4). There was an association between eggs and diet for all the strains (Table 5). Similarly, Xiaoyong indicated the significant effect of the Co concentration of diet on the Co concentration of eggs (Lujiang et al., 1996).

**Mn**: As stated in Tables 3 and 4, the highest concentration of Mn was 6.52 mg/kg in the egg yolk of the native strain, whereas Hi‐line egg white had the lowest concentration (0.035 mg/kg fresh weight). Demirulus (2013) reported a value of 1.9 mg/kg in the yolk. Mn concentration of feed is summarized in Table 5. Mn concentration of the egg in all the strains was significantly affected by Mn in the diet (*p*‐value <0.001). Venglovska et al. (2014) showed 1.65 mg/kg Mn in dry matter of egg yolk of the Lohman brown strain and, also, 46.4 mg/kg of Mn in the basal diet as well as reported that feed supplementation significantly influenced yolk Mn concentration.

Table 5 describes the mean concentration of essential toxic metals in feed in five strains of poultry hens. Furthermore, all the five‐type assessed feed samples exhibited the following order for metal concentrations: Fe > Mn > Zn > Cu > Se > Co, except for Highline feed samples which contained higher Co content than non‐metal Se. Total egg content of metal concentrations was according to the following order as well: Fe > Zn > Mn > Cu > Se > Co. Generally, a major difference (*p*‐value <0.001) was demonstrated among the concentration of metals of the feed in different strains. Therefore, as estimated, the diet had the highest effect on the trace element concentration in egg (Van Overmeire et al., 2009; Vincevica‐Gaile et al., 2013). As it can be observed, native hens had the lowest Zn content in the feed, and the highest Zn content in the egg white and yolk, showing the significant effect of strain on Zn content, which might be due to the genetically differences leading to differences in feed consumption patterns, utilization and pattern of fat storage and different absorption patterns of micronutrients (Vincevica‐Gaile et al., 2013). Table 6 shows the correlation between metals in egg white, egg yolk and feed. Moreover, a significant correlation among most trace elements in egg white, yolk and the whole egg was observed. But there was no level of significance among metals in the diet despite Zn and Mn.

**TABLE 6:** The correlation of trace elements concentration in egg white, yolk and total eggs.

Part of egg	Metal	Se	Fe	Zn	Co	Mn
White egg	Cu	0.556^a^	0.695^a^	0.763^a^	0.416^a^	0.597^a^
	Se		0.327^b^	0.431^a^	−0.135	0.252
	Fe			0.727^a^	0.606^a^	0.692^a^
	Zn				0.435^a^	0.896^a^
	Co					0.409^a^
Yolk egg	Cu	0.375^a^	0.661^a^	0.724^a^	0.270	0.633^a^
	Se		0.035	0.487^a^	−0.042	0.516^a^
	Fe			0.528^a^	0.005	0.392^a^
	Zn				0.214	0.942^a^
	Co					0.380^a^
Whole egg	Cu	0.798^a^	0.857^a^	0.913^a^	0.283^a^	0.863^a^
	Se		0.645^a^	0.784^a^	0.026	0.728^a^
	Fe			0.859^a^	0.287^a^	0.816^a^
	Zn				0.287^a^	0.975^a^
	Co					0.334^a^
Feed	Cu	−0.400	0.300	−0.300	0.300	−0.100
	Se		−0.400	−0.500	−0.500	−0.800
	Fe			0.700	0.800	0.600
	Zn				0.500	0.900^b^
	Co					0.600

^a^ Correlation is significant at the 0.01 level (2‐tailed).

^b^ Correlation is significant at the 0.05 level (2‐tailed).

#### 3.1.1 Health risk assessment of egg

To assess the potential impacts of contaminants on human health, a risk assessment is used. To evaluate the non‐cancer risks associated with food consumption (for children and adults), THQs were used. THQ is the ratio of the detected quantity of a pollutant to a standard reference dose level. The exposed population will likely suffer adverse effects if they expose to higher doses (Wang et al., 2005). This methodology does not provide a quantitative estimate of the probability of an exposed population experiencing a reverse health effect. But it shows a warning of the risk level due to contaminant exposure (Shaheen et al., 2016). Table 7 shows the mean values of THQs (dimensionless, THQs), standard deviations and *p*‐values for Cu and Zn in Children and Adults according to different strains. Table 7 reveals that almost all the amount of THQs of Zn and Cu was less than even 0.1. Therefore, the resident population was not experiencing the adverse effect caused by the consumption of eggs. Our results were in accordance with the study of Shaheen et al. (2016). In the case of children, regarding that they are more sensitive to harmful effects, this point is described in formulas by nearly half their weight, and more attention is needed. Although their THQs values were higher than that of adults, they were still in a safe range (Wang et al., 2005). There was a significant impact of strain on THQs in children and adults (*p*‐value = 0.000, Table 7).

**TABLE 7:** Mean values of target hazard quotients (THQs) (dimensionless, THQs), standard deviations and *p*‐values for Cu, Zn in children and adults according to different strains.

THQ value		N	Mean	Std. deviation	*p*‐Value
THQ Cu, adults	Native	20	0.013	0.010	0
	Bovans	20	0.019	0.015	
	LSL	20	0.013	0.006	
	Shaver	20	0.008	0.003	
	Hi‐line	20	0.004	0.006	
	Total	100	0.011	0.010	
THQ Cu, children	Native	20	0.029	0.023	0
	Bovans	20	0.042	0.033	
	LSL	20	0.030	0.014	
	Shaver	20	0.018	0.007	
	Hi‐line	20	0.008	0.013	
	Total	100	0.025	0.023	
THQ Zn, adults	Native	20	0.068	0.082	0
	Bovans	20	0.064	0.051	
	LSL	20	0.028	0.015	
	Shaver	20	0.023	0.015	
	Hi‐line	20	0.008	0.014	
	Total	100	0.038	0.050	
THQ Zn, children	Native	20	0.152	0.182	0
	Bovans	20	0.143	0.114	
	LSL	20	0.062	0.032	
	Shaver	20	0.050	0.033	
	Hi‐line	20	0.019	0.031	
	Total	100	0.085	0.111	

## 4 CONCLUSION

The current study assessed the effect of strain and diet on egg essential elements and surveyed the health risk of them. All the five essential elements had the lowest values in the Hi‐line strain in both yolk and white and, consequently, in the whole egg. As stated in the Scheideler study, perhaps the Hi‐line strain could have a more effective use of the diet. The potential health risk assessed by the THQs methodology was verified by US EPA, and their values were below one showing a safe range, but if all the trace elements take into account (sum of individual metal's THQs, the TTHQ), more than one TTHQ is probable. In addition, if the trace elements of the other major protein sources like meat and milk are considered too, probably the combined Hazardous Index (sum of TTHQs of different kinds of stuff) exceeds the acceptable limit, which may be a concerned authority on the potential health risks. All the amount of THQs of Zn and Cu was less than even 0.1. It is strongly recommended to evaluate the trace elements from food sources in the diet.

## AUTHOR CONTRIBUTIONS

*Investigation; writing – original draft*: Amin Azadi. *Conceptualization; methodology; writing—original draft*: Majid Aminzare. *Data curation; investigation; writing – original draft; writing – review and editing*: Seyyed Mohammad Ali Noori.

## CONFLICT OF INTEREST STATEMENT

No potential conflict of interest was reported by the authors.

## FUNDING INFORMATION

This study was supported financially by Ahvaz Jundishapur University of Medical Sciences, Ahvaz, Iran (grant no: U‐98004).

### ETHICS STATEMENT

This study was approved by ethical committee of Ahvaz Jundishapur University of Medical Sciences, Ahvaz, Iran (IRAJUMS.REC.1398.100).

### PEER REVIEW

The peer review history for this article is available at https://publons.com/publon/10.1002/vms3.1184.

## Data Availability

No.
